# Feasibility and acceptability of the use of flash glucose monitoring encountered by Indigenous Australians with type 2 diabetes mellitus: initial experiences from a pilot study

**DOI:** 10.1186/s12913-023-10121-6

**Published:** 2023-12-08

**Authors:** Audrey Sing Yi Eer, Rebecca Chia Yee Ho, Tracey Hearn, Mariam Hachem, Megan Freund, Luke James Burchill, Sharon Atkinson-Briggs, Satpal Singh, Sandra Eades, Richard Charles O’Brien, John Stuart Furler, David Norman O’Neal, David Andrew Story, Jeffrey David Zajac, Sabine Braat, Alex Brown, Phillip Clarke, Ashim Kumar Sinha, Anna Gerardina McLean, Stephen Morris Twigg, Elif Ilhan Ekinci

**Affiliations:** 1 Austin Health, Heidelberg, VIC Australia; 2 The University of Melbourne (Austin Health), Melbourne, VIC Australia; 3 Albany Health Campus, Spencer Park, WA Australia; 4 The University of Melbourne, Melbourne, VIC Australia; 5 Rumbalara Aboriginal Co-Operative, Mooroopna, VIC Australia; 6 Centre for Research and Education in Diabetes and Obesity (CREDO), Faculty of Dentistry Health Sciences and Medicine, The University of Melbourne, Austin Health, Melbourne, Australia; 7 The Australian Centre for Accelerating Diabetes Innovation (ACADI), The University of Melbourne, Parkville, Australia; 8 Research Academic, School of Medicine and Public Health, University of Newcastle, Newcastle, NSW Australia; 9 Equity in Health and Wellbeing Research Program, Hunter Medical Research Institute, New Lambton Heights, NSW 2305 Australia; 10 Royal Melbourne Hospital, Parkville, VIC Australia; 11 Department of Medicine (Royal Melbourne Hospital), Aboriginal Cardiovascular Health Equity Research Group, The University of Melbourne, Melbourne, VIC Australia; 12 Department of Medicine, The University of Melbourne, Melbourne, Australia; 13 Faculty of Medicine Dentistry and Health Sciences, The University of Melbourne, Melbourne, VIC Australia; 14 Centre for Epidemiology and Biostatistics, Melbourne School of Population and Global Health, The University of Melbourne, Melbourne, VIC Australia; 15 Austin Clinical School, The University of Melbourne, Melbourne, VIC Australia; 16 Graduate Programs and Executive Education, Melbourne Medical School, The University of Melbourne, Melbourne, VIC Australia; 17 Lipid Services, Austin Health, Heidelberg, VIC Australia; 18 Department of General Practice, Faculty of Medicine Dentisty and Health Sciences, The University of Melbourne, Melbourne, VIC Australia; 19 St. Vincent’s Hospital Melbourne, Fitzroy, VIC Australia; 20 The University of Melbourne (St. Vincent’s Hospital), Melbourne, VIC Australia; 21 Department of Critical Care, The University of Melbourne, Melbourne, Australia; 22 Melbourne Academic Centre for Health (MACH), Melbourne, VIC Australia; 23 Division of Medicine, Medical Services CSU and Department of Endocrinology, Austin Health, Heidelberg, VIC Australia; 24 MISCH (Methods and Implementation Support for Clinical Health) research Hub, Faculty of Medicine, Dentistry and Health Sciences, The University of Melbourne, Melbourne, Australia; 25 Indigenous Genomics, Australian National University and Telethon Kids Institute, Canberra, Australian Capital Territory Australia; 26 Health Economics, Nuffield Department of Public Health, Univeristy of Oxford, Oxford, UK; 27 Academic, Melbourne School of Population and Global Health, The University of Melbourne, Melbourne, VIC Australia; 28 Diabetes and Endocrinology, Cairns Hospital, Cairns, QLD Australia; 29 James Cook University, Cairns, QLD Australia; 30 Endocrinology and General Medicine, Cairns Hospital, Cairns, QLD Australia; 31 Menzies School of Health Research, Darwin, NT Australia; 32 Department of Endocrinology, Royal Prince Alfred Hospital, Camperdown, NSW Australia; 33 Endocrinology, Stan Clark Chair in Diabetes, Faculty in Diabetes, The University of Sydney, Sydney, NSW Australia; 34 Sir Edward Weary Dunlop Principal Research Fellow in Metabolic Medicine, University of Melbourne, Melbourne, VIC Australia

**Keywords:** Indigenous Australian, Aboriginal people, Type 2 diabetes mellitus, Flash glucose monitoring, Qualitative research, Phenomenological study

## Abstract

**Background:**

Type 2 diabetes mellitus (T2DM) is highly prevalent within the Indigenous Australian community. Novel glucose monitoring technology offers an accurate approach to glycaemic management, providing real-time information on glucose levels and trends. The acceptability and feasibilility of this technology in Indigenous Australians with T2DM has not been investigated.

**Objective:**

This feasibility phenomenological study aims to understand the experiences of Indigenous Australians with T2DM using flash glucose monitoring (FGM).

**Methods:**

Indigenous Australians with T2DM receiving injectable therapy (*n* = 8) who used FGM (Abbott Freestyle Libre) for 6-months, as part of a clinical trial, participated in semi-structured interviews. Thematic analysis of the interviews was performed using NVivo12 Plus qualitative data analysis software (QSR International).

**Results:**

Six major themes emerged: 1) FGM was highly acceptable to the individual; 2) FGM’s convenience was its biggest benefit; 3) data from FGM was a tool to modify lifestyle choices; 4) FGM needed to be complemented with health professional support; 5) FGM can be a tool to engage communities in diabetes management; and 6) cost of the device is a barrier to future use.

**Conclusions:**

Indigenous Australians with T2DM had positive experiences with FGM. This study highlights future steps to ensure likelihood of FGM is acceptable and effective within the wider Indigenous Australian community.

**Supplementary Information:**

The online version contains supplementary material available at 10.1186/s12913-023-10121-6.

## Introduction

Type 2 diabetes mellitus (T2DM) is a highly prevalent disease affecting approximately 1 million adults in Australia [1]. Indigenous Australians are affected at a disproportionately higher rate compared with non-Indigenous Australians, with the former being three times more likely to have T2DM [2]. Suboptimally managed T2DM leads to complications that increase mortality and morbidity [3]. This is of particular concern for Indigenous communities [4–7].

Current approaches of capillary blood glucose monitoring are associated with pain, inconvenience, social stigma and needle phobia [8]. One of the recent advances in the self-management of diabetes has been the ability to use interstitial fluid glucose to measure glucose levels. This novel glucose monitoring technology tracks interstitial glucose in near real-time, providing users with not only information on glucose levels but trends which can aid diabetes management. Currently, two categories of these devices are available to people with diabetes; real time-continuous glucose monitoring (RT-CGM) [9] and intermittent flash glucose monitoring (FGM) [10]. Additionally, there are devices that provide retrospective CGM (Medtronic iPro) and FGM (Abbott Freestyle Libre Pro), with data aimed at health professionals rather than people living with diabetes. Qualitative research of CGM in T2DM suggests this technology allows users to visualise glucose levels, facilitating lifestyle (dietary and exercise) decisions in response to their visualised glucose levels and patterns [11]. At the time of writing, these RT-CGM devices available in Australia rely on capillary glucose for calibration. FGM devices in contrast, are factory-calibrated. However FGM devices in Australia did not at the time offer the alarms for hypo- or hyperglycaemia available on CGM devices, unless the user scans the sensor.

While there is evidence that FGM improves diabetes management compared to usual care [12], a recent meta-analysis did not find FGM to be superior to CGM [13]. Neither of these studies focused on Indigenous people with T2DM.

Given the potential benefits of FGM, further research is needed to determine whether FGM is feasible and acceptable for Indigenous Australians living with T2DM, in regional locations. A qualitative research approach was used to address this question with a view to providing insight to guide future research protocols and utilisation of FGM devices in Indigenous communities. As such, a qualitative study was undertaken that aimed to explore the feasibility, acceptability and experiences of Indigenous Australians living with T2DM using FGM.

## Methods

### Study setting and design

This feasibility phenomenological research was nested within a 6-month, non-blinded, randomised control trial (RCT) pilot study comparing the effectiveness of FGM to conventional self-monitored blood glucose (SMBG) with capillary blood glucose on HbA1c levels, and time spent in hyper- and hypoglycaemia. This study was undertaken at Rumbalara Aboriginal Co-operative (Mooroopna, Victoria, Australia) and reported according to the Consolidated Criteria for Reporting Qualitative Studies (COREQ) [14]. Rumbalara Aboriginal Co-operative is an Aboriginal Community-Controlled Health Organisation in the Greater Shepparton region of Victoria, approximately 180 km north of Melbourne. All participants provided written informed consent. The study was approved by the Goulburn Valley Health Human Research Ethics Committee. The research was conducted with respect for Indigenous peoples and communities’ shared values, diversity, priorities, needs and aspirations with the intention of benefiting Indigenous people and communities as well as researchers [13]. The study was conducted with the understanding to enhance the rights of Indigenous peoples as researchers, research partners, collaborators and participants in research [15].

**Fig. 1:**
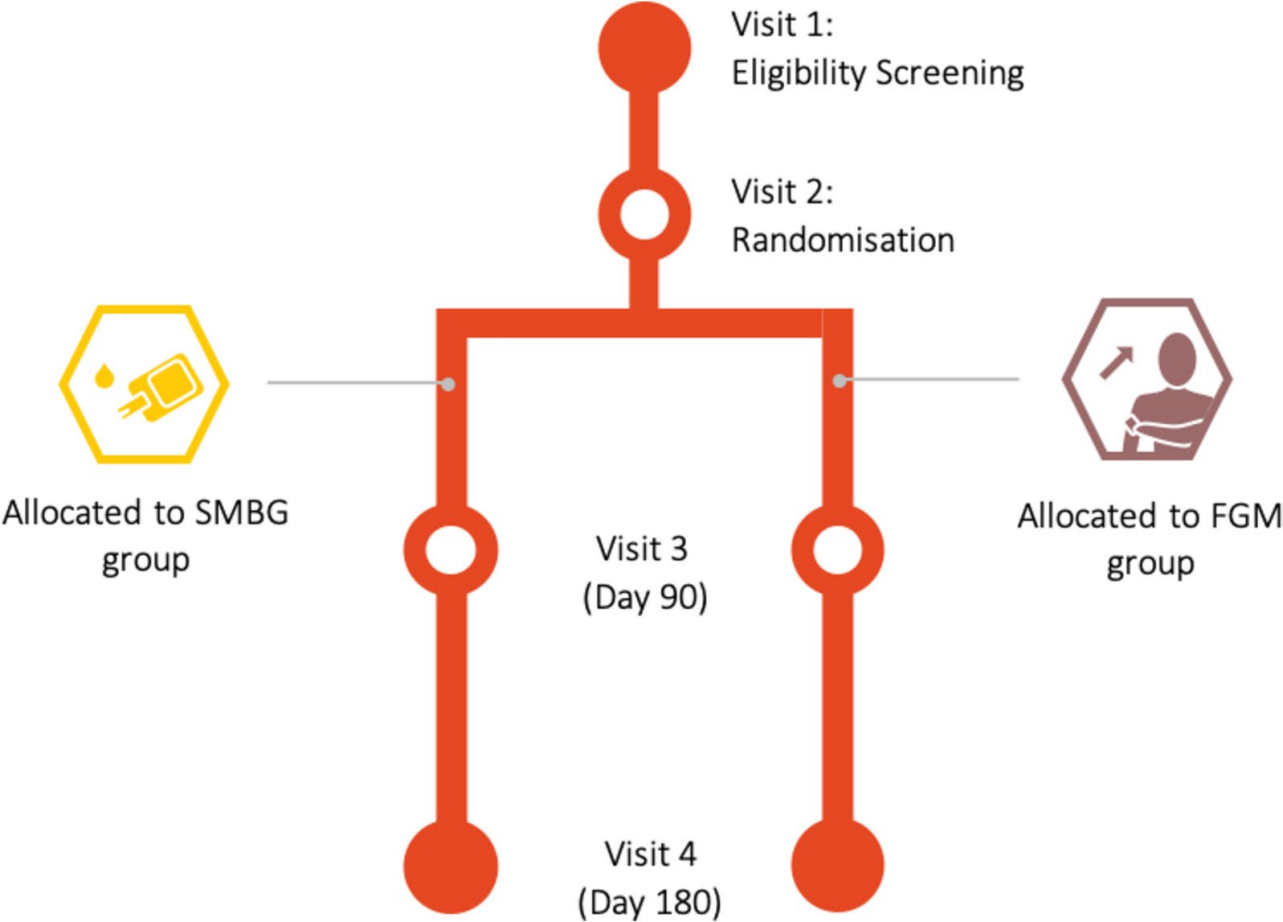
The study design for the 6-month RCT comparing FGM to SMBG. During visit 1, participants were screened to see if they met the inclusion or exclusion criteria. At visit 2 randomisation occurred. Follow up was then organised 3 months later (visit 3) and 6 months later (visit 4)

Potential participants were identified from patient databases or during study visits from the overarching RCT, for which recruitment is still underway. Participants were included in the RCT if they met the inclusion criteria outlined in Table 1. During visit 1 (Fig. 1), baseline participant information regarding age, sex, diabetes duration, comorbidities, living arrangements and remoteness were collected. Participants’ diabetes therapy was recorded, weight, height, blood pressure and heart rate were measured, and blood and urine tests were performed. Blinded CGM (Medtronic iPro2) was worn for one week and collected at visit 2, when participants were randomised into either usual care (SMBG) or FGM. The blinded CGM was used to measure time spent in target glucose, low glucose and high glucose, and baseline data had no impact on treatment allocation. The Medtronic iPro2 was used for blinded CGM as the Freestyle Libre Pro blinded CGM was not yet available for use. As part of the 6-month RCT, participants randomised to the usual care arm were offered FGM devices for 6 months after the conclusion of the study.

**Table 1:** Inclusion and exclusion criteria for the 6-month RCT comparing FGM to SMBG

**Inclusion Criteria**
Indigenous Australian
T2DM
≥ 18 years old
HbA1c ≥ 53 mmol/mol (7%)
Injectable hypoglycaemic therapy
• Insulin and/or
• GLP-1 analogue
**Exclusion Criteria**
T1DM
< 18 years old
Known allergy to medical-grade adhesives
Active illicit drug or heavy alcohol use (> 4 standard drinks/day)
Chronic kidney disease requiring dialysis
Active malignancy
Receiving varying doses of corticosteroid therapy
Amphetamines, anabolic or weight reducing medications or about to undergo bariatric surgery
Pregnancy
Cognitive impairment preventing instructions to be followed

### FGM procedures in pilot RCT

Participants were shown how to insert the device into the underside of their arm, in accordance with the manufacturer’s instructions. Participants were advised that the sensors could provide glucose data for up to 14 days, and needed replacement after 14 days. Participants were instructed to scan the sensor with a FGM reader that captured the glucose data. They were advised to scan the sensor at a minimum of at least every 8 h, because the reader captured the preceding 8 h of glucose data with each scan. They were advised there was no maximum limit to scanning, to ensure maximal glucose data was captured. Participants initially only had the option of using the Freestyle Libre reader to scan the FGM sensor as the LibreLink smartphone application had not yet become available for commercial use in Australia. Amendments were later made to enable participants to use their smartphone with the LibreLink application when this became available. Lastly, an explanation of how to interpret the data was provided, including the meaning of the arrow visible on the device screen and how to interpret the graphical data in relation to blood glucose readings.

### Participant recruitment for the qualitative study

**Fig. 2:**
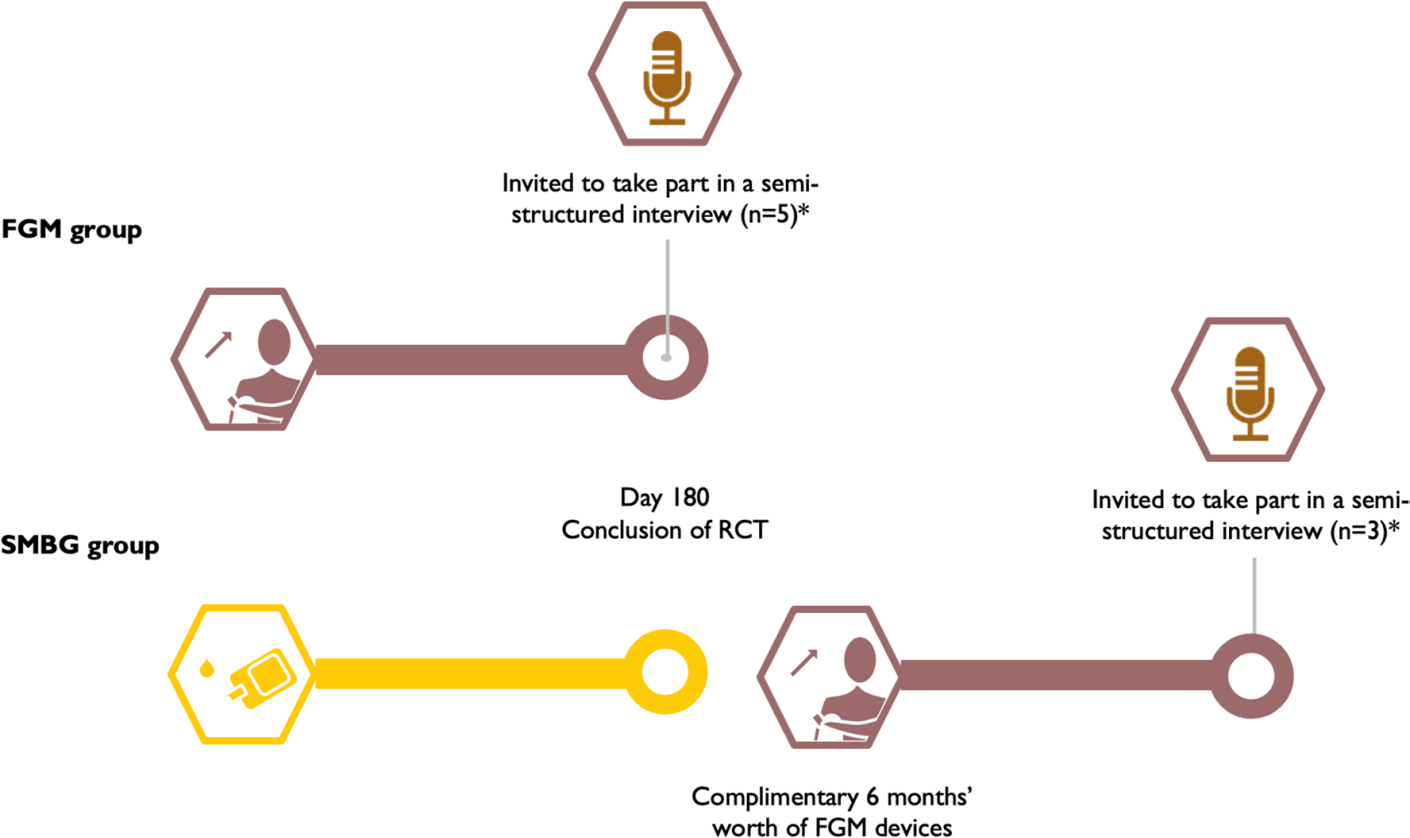
As part of the 6-month RCT, participants who were randomised to the SMBG arm were given 6 months’ worth of FGM devices to use at the conclusion of the study as an appreciation for participating. Participants who took part in the RCT who had completed 6 month use of FGM were then invited to take part in a semi-structured interview from both the FGM and SMBG group. *At the time of this study, eight participants had completed the RCT and accessed 6 months of FGM. All eight participants completed the semi-structured interviews. Five participants were from the FGM arm (after 180 days of FGM) and 3 from the SMBG arm (after 180 days of FGM following the initial 180 days of SMBG)

Participants from both the FGM and SMBG groups who completed 6 months use of the FGM device were invited to participate in the qualitative study (Fig. 2). Semi-structured, one-on-one interviews discussing participant experiences of FGM were conducted at Rumbalara Aboriginal Co-operative. The study profile is displayed in Fig. 1.

### Qualitative data collection

Interviews were conducted at Rumbalara Aboriginal Co-operative medical clinic in a private room. All interviews were facilitated by a single investigator (RH), a non-Indigenous Australian female. RH was part of the RCT team and had contact with participants prior to conducting the semi-structured interviews. The interviews explored the acceptability of the device, the influence the device had on the participants’ life and the role the device may have in the family. Broad, open ended questions were used, and clarification of any topics were attempted as required. All interviews were audio-recorded in the presence of the interviewer and interviewee only.

### Qualitative data analysis

Audio-recordings of all interviews were transcribed verbatim by RH. All identifying text were removed to protect participants’ confidentiality. From these transcripts, the text was analysed using the NVivo12 Plus program [8]. Inductive thematic analysis was used to code and identify key words and phrases. Themes were developed separately by two investigators (TH and RH). Similarities and differences were identified and grouped according to emerging themes. Investigator TH was a local Yorta Yorta woman whose involvement in analysis enabled correct interpretation of the text. For any differences that arose, a third investigator (AE) was involved to discuss and resolve any discrepancies until consensus was reached.

## Results

### Participants

This study took place whilst the pilot RCT exploring FGM in Indigenous Australians with T2DM was undergoing recruitment. At the time of this study, eight participants had completed the RCT and accessed 6 months of FGM. All eight participants completed the semi-structured interviews. Five participants were from the FGM arm and 3 from the SMBG arm. Participants were interviewed about their experiences using the FGM device. The duration of the interviews ranged between 9 and 18 min.

The median age of participants was 56 years (range 35–75 years) with a median duration of diabetes of 14.5 years (range 4–25 years) and median HbA1c level of 69 mmol/mol (range 54–99 mmol/mol) (8.5%, range 7.1 – 11.2%) (Table 2). Seven (88%) participants were taking metformin as part of their hypoglycaemic therapy and all used insulin.

**Table 2:** Participant demographic data at baseline, prior to using the FGM device

Participant demographic data	Statistics (*n* = 8)
Age (years)	56 (35–75)
Sex, n(%)	
Male	5 (62)
Female	3 (38)
Duration of diabetes (years)	14.5 (4–25)
BMI (kg/m^2^)	40.0 (25.0–52.2)
HbA1c (mmol/mol)	69 (54–99)
HbA1c (%)	8.5 (7.1–11.2)
Glycaemic Therapy, n(%)	
Metformin	7 (88)
SU	2 (25)
SGLT2i	3 (38)
DPP4i	1 (13)
GLP-1	3 (38)
Insulin	8 (100)

Data is presented as n(%) and median (range: minimum to maximum) unless otherwise stated

*BMI* Body mass index, *HbA1c* glycated haemoglobin, *SU* sulfonylurea, *SGLT2i* sodium-glucose transporter-2 inhibitor, *DDP4i* dipeptidyl peptidase-4, *GLP-1* glucagon-like peptide-1

### Findings

**Fig. 3:**
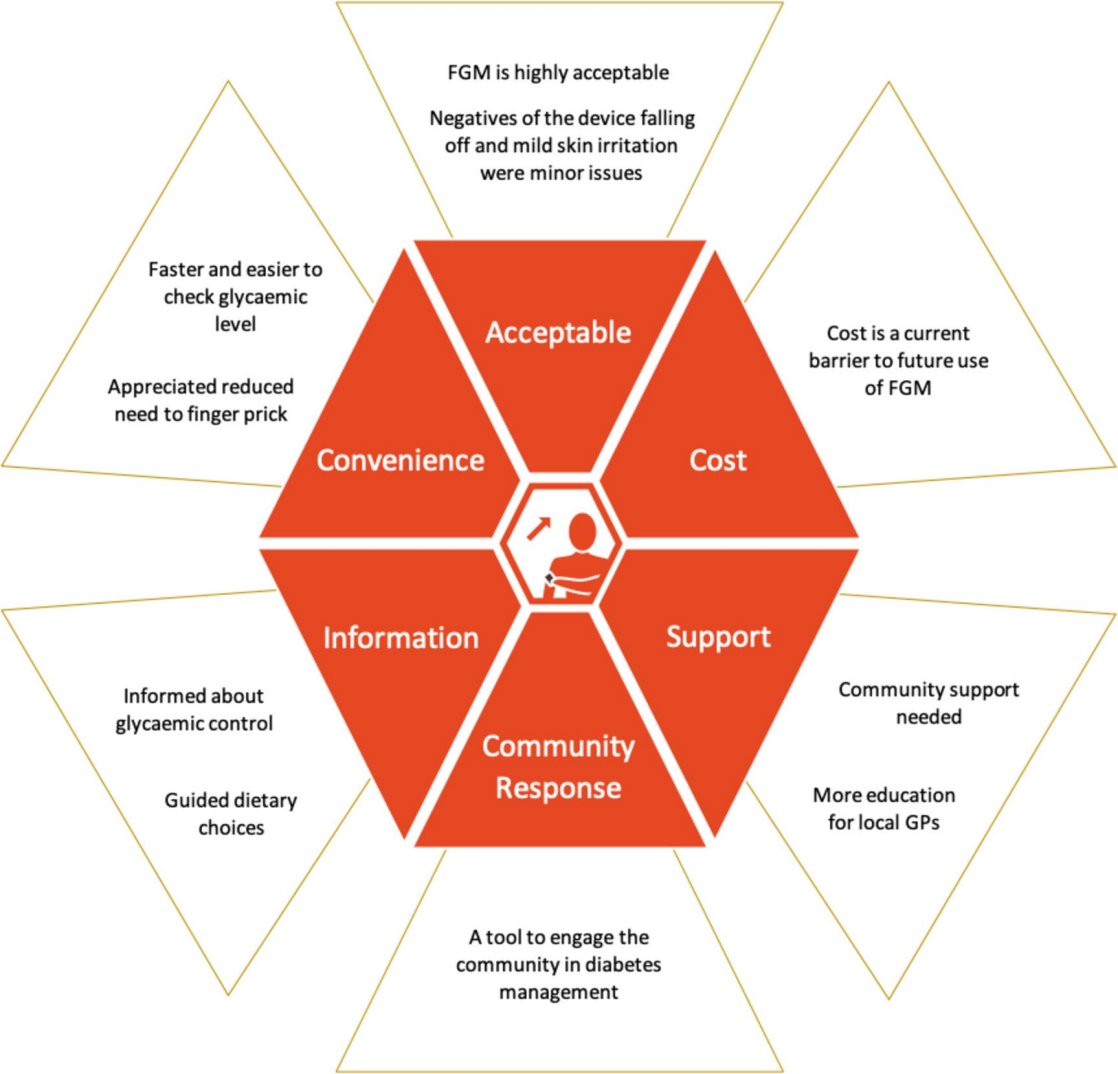
Experiences and opinions of the use of FGM. Themes and subthemes that emerged from semi-structured interviews with participants about their experience using the FGM device

From the data collected, six major themes emerged: 1) FGM was highly acceptable to the participant; 2) the perceived primary benefit of FGM was convenience of using the technology to measure glucose 3) data from FGM acts as a tool to modify lifestyle choices; 4) FGM needs to be complemented with health professional support; 5) FGM could be a tool to engage communities in diabetes management; and 6) the cost of the device is a barrier to future use. This thematic series is summarised in Fig. 3 and the complete set of quotes obtained is available in Appendix 1. No major differences were identified between participant responses from those in the FGM and SMBG groups.

### Theme 1: FGM is highly acceptable

Overall, participants had positive experiences of FGM. Participants suggested that it easily fit within their lifestyle and did not interfere with their day-to-day lives.

*“at first you knew that there was something there, but then you got use to it, it just grew with you and it become a part of you, with your diabetes, which was good” (P6).*

Others spoke of how you would “*forget that it’s there” (P8)* or how “*it fits well, don’t even know it’s there” (P3).*

Participants noted some issues with the sensor but considered these issues minor in comparison to the benefits FGM provided. These included the sensor being knocked/falling off or mild skin irritation.

*“just got to be careful where you lay and because you’ll get caught onto something and that something can just pull it off” (P8).*

### Theme 2: Convenience of FGM as a primary benefit identified

Participants discussed the ease of checking glucose readings with FGM compared to SMBG. This was particularly noticed when participants were socialising.

*“If you were out and something, you just go ‘wave’ whereas you got to sit somewhere and you know prick your finger and monitor and you’ve got everyone watching whereas that’s [FGM] more discreet”(P4),*

Another participant talked about how when driving *“it wasn’t a hassle of getting that bag and doing all that other stuff, it was just a matter of just scanning it and keep going” (P5).* Or that the FGM is *“just much more handy you know, having to only carry around that [reader] instead of the pouch that’s got the machine, the needles, it’s got the strips and all that” (P7).*

Participants’ subjective reporting indicated they were more likely to check their glucose readings compared to capillary glucose monitoring. A participant stated that “*I was more inclined to use it [FGM], I don’t use the finger-prick much at all, but I did use that [FGM] one” (P4).* Others said *“definitely found it much easier and good to take with me you know especially at work where I could just use it at lunch time” (P5)* or that *“I could monitor my sugar anytime I wanted” (P1)*.

Furthermore, in comparison to SMBG, there was an appreciation that participants no longer had to rely heavily on capillary glucose testing to monitor glycaemic levels. One participant stated:

*“I’m glad that I didn’t have to prick myself every day. I’m glad that you can just scan it. That’s the best thing that ever happened to me” (P8).*

### Theme 3: FGM data as a tool to modify lifestyle choices

The data provided by FGM was beneficial to most participants. One participant expressed that she *“was informed” (P4).* In particular, comments were made regarding how the data from the FGM device specifically helped inform dietary choices.

*“A better way of looking at it. You know what I mean? Especially if you’re going low and stuff, and too high, you’re like whoop, what did I have, what did I eat, what did I drink, that kind of thing” (P7).*

Others also stated:

*“…makes you think about what you put in your mouth because it does show up and you can’t dodge no bullets, you can actually see it” (P4).*

The accountability and visual cue that the device provided appeared to be the driving factor for dietary changes. Others responded in a similar manner:

*“It stopped me eating a bit. So that was one thing about it. I was watching my food intake*” *(P2)* and “*I thought about what I ate a lot better and I’d knock things back” (P1)*. *“You’re constantly looking at the readings and then I think “oh, I’ve got good readings so I’m doing the right thing, so yeah it does, it does change what you eat”* (P5).

The visual data the FGM device provides could be well suited to the Indigenous Australian population. One participant said “*Indigenous people are actually better at seeing things, it’s got more of an impact I think*” *(P4)* when talking about the data represented as a graph.

On the other hand, the information provided may not be interpreted by all users. One participant stated that *“I don’t understand it [the data] at all, but I know that it’s you know, it’s for professional people to read it” (P3).*

### Theme 4: FGM needs to be complemented with health professional support

Participant experiences may suggest that the use of FGM is helping users to stay engaged with their healthcare teams. It appeared that some participants, P1 (73 years), P3 (75 years), P8 (50 years) relied on health professional staff to replace their FGM sensor every 2 weeks. One participant said *“I could probably do it but yeah, just come in and have a chat” (P3).* While another talked about how “*everybody worked very very well together and supported me which is wonderful” (P1)* when talking about the diabetes nurse educators and aged care nurses who helped replace the sensor.

Furthermore, other participants said that being part of the study, they found that “*it was good to have support, people that are around that communicate and help” (P6)* and they said *“thank you for giving me the opportunity anyway and being support to me as well.” (P5).*

There may also be a role in further education and support for local general practitioners (GPs), with one participant stating, “*first time they’d [GP] seen it*” *(P2)*. Another participant said that they *“normally leave it up to the professional people” (P3).*

### Theme 5: FGM could be a tool to engage communities in diabetes management

When participants were asked about how others in their community reacted to seeing them use the FGM device, the feedback was positive:*“‘Ooh that’s good, that’s cool.’ So yeah they all liked it” (P4).* Some even had others ask how they themselves could access the technology, *“I think they’re fascinated by the technology of reading their sugars and the ones which have diabetes, ‘where’d you get that, where’d you get that, how’d you get that?’”(P3).*

Despite many within the community not having previously known about the new technology, there was support for its use:

“I’ve never seen anything like that but it was just a new device and they, most people understood, diabetes and that… they say you know, they should bring out more technology or something for diabetes” (P5).

One participant stated that *“everyone was like, check your sugar, check your sugar.” (P7).*

This included a young boy in her care:

*“I’d have the little bloke...he thinks it’s cool, so he’s always on my back, checking, checking, checking” (P7).*

The possibility that the visibility of wearing the device could have a negative impact was also raised. A participant raised the idea that “*people do judge you, because you’re Aboriginal, you’ve got diabetes*” *(P5).* She felt that *“it’d depend on if other people seen that I’ve got it”* if wearing the device would feed into that judgement.

### Theme 6: Cost as a current barrier to future use of FGM

Participants received the FGM device free of charge as part of the study. However, many participants were aware that the consumable component of the system costs roughly $100 to replace every fortnight. Participants stated that “*it’s a good thing to have, but paying for it, no. Not a hundred dollars a go” (P2).* Another participant nearing completion of using the device said *“if it was cheaper, I’d continue to get it you know, but I just don’t know that spending that every fortnight you know…might hurt the budget a little” (P7).* One participant hoped that the government would help fund the device, “*…but it’s not cheap. It’s expensive. But it’s a new program and it’s really helpful. So I hope the government will continue to help and support people with high sugar” (P1).*

## Discussion

This is the first study on the use of FGM in the Indigenous Australian population. The findings demonstrated that Indigenous Australians living with T2DM who participated in the RCT, had positive experiences with FGM. Participants reported greater awareness of glycaemic levels resulting in healthier dietary choices. Widespread community support suggests a role for FGM in engaging the broader Indigenous community in diabetes care. Barriers to implementation identified in this study include affordability, stigma associated with being Aboriginal and living with diabetes, and the need for community support and education.

A major benefit from this novel glucose monitoring technology is reduced time spent in hypoglycaemia [16–18]. Most participants stated that despite being on insulin therapy hypoglycaemia was not common and hyperglycaemia was the predominant issue. Whilst this was a small feasibility study, a subtheme that emerged from the qualitative data was that FGM may influence dietary choices which may have a positive impact on glucose levels in people with T2DM. This FGM data empowered participants with self-reliance that food choices and exercise could help them manage their diabetes. Alternatively, the FGM data can help healthcare practitioners to intensify diabetes therapies as they have greater confidence in detecting and minimising hypoglycaemia. Compared to FGM, SMBG provides less glucose data which is a barrier to both initiating and intensifying insulin therapy [19, 20].

Some studies have shown that the data from FGM can lead to improvements in glycaemic management in people with T2DM independent of changes in diabetes medications [11, 21, 22]. In particular users have described dietary changes made as a result of knowledge gained from CGM data [11]. This benefit was seen in participants in this pilot study, but the potential issue of not understanding the data presented was also raised. This suggests that the use of FGM may need to be accompanied with comprehensive education to ensure maximal benefit from FGM is obtained. Future studies could be performed to further explore this challenge to guide how to best equip users for newer technologies without leaving them feeling inundated with a vast amount of data. The education required would be both: (i) real-time monitoring to understand glucose levels, trend arrows and appropriate self-care at the time, and [23] appreciating the content of the summary reports of the Ambulatory Glucose Profile (AGP), especially time in range, and time spent in hypoglycaemia [24].

Similarly, this study raised concerns that local health practitioners may not have adequate exposure or experience interpreting and acting on data provided by FGM. As more and more people begin to use this novel technology this may change. However, to ensure that the most cost-effective outcome from FGM is achieved, formal training could be provided to local GPs and health practitioners to help improve further glycaemia management.

For some participants the use of FGM provided opportunities for more regular contact with health practitioners due to the need for the sensor to be replaced every 14 days. While some participants did this independently, others preferred their health practitioner to replace it for them. Despite stating they could have done it by themselves, they enjoyed the support and social nature of having it replaced by someone else. This increased regular contact with health services could be a confounding factor to improvements in glycaemic management attributed to FGM use. However close contact with health services through use of FGM may also be a way to increase the participant’s engagement with their diabetes management.

The positive responses from other Indigenous community members on individuals using FGM both contributes to the acceptability of this novel device, but also may indicate the potential use of this technology as a tool for engaging the wider community in diabetes management. The community support of the device has been encouraging. The Indigenous community in the Greater Shepparton region are closely connected and the way others respond positively to individuals using the device supports the idea that FGM could be utilised in the future. This is important as previous implementation of health technologies in Indigenous Australian populations have demonstrated that community awareness of technology influences acceptability, as demonstrated in a qualitative study of a mental health app designed for use by Indigenous Australians [25]. The novelty and ease of the FGM device means that diabetes management can be shared amongst an individual’s family, friends and community rather than solely by the individual with T2DM. It could be inferred that the device could act as a vessel to promote community awareness of diabetes, something that can be explored in further studies. This would be critical as previous studies have demonstrated that community engagement and education are crucial to an effective intervention [26–28]. Further studies could explore the effect that the introduction of FGM has on the wider community to assess a secondary benefit that might not be evident from studies at the individual level.

The issue that visibility of the FGM sensor may draw unwanted stigma towards those wearing it was raised. How this affects the success of the implantation of FGM is hard to currently predict. It will depend on how the general public view the device and diabetes itself.

### Limitations

Most participants only used the Freestyle Libre reader rather than a smartphone with the LibreLink application to check their glucose levels, as the smartphone application was not initially available for commercial use. This had both its advantages and disadvantages. Firstly not everyone in the community had a compatible smartphone, so the use of the reader did not disadvantage those without a smartphone. However if FGM users forgot to bring their reader to appointments, their glucose data was not available for review by their clinician. In contrast the smartphone application with its cloud connectivity could alleviate the dilemma of forgotten readers, but also has its own challenges with internet connectivity and software requirements. Furthermore now that the Freestyle Libre data is stored on the cloud, whilst it should be secure, there is always the risk of data breaches. Also the Freestyle Libre 1 sensor was used in this study as the Freestyle Libre 2 sensor, which has additional features such as predictive hypoglycaemia alarms, was not yet commercially available. Whilst the features of the improved FGM sensors may reduce time in hypoglycaemia, the alarms could cause more anxiety and diabetes distress.

Continuous and flash glucose monitoring is expensive and this is a barrier to its use. In Australia, CGM use is subsidised for use by people with type 1 diabetes, but there is no subsidy for people with type 2 diabetes, Indigenous people or other high risk groups. This pilot RCT is assessing the feasibility of performing a larger multicentre RCT using FGM in Indigenous Australians with type 2 diabetes. It is hoped that future studies will provide evidence to support this technology being subsidised for use by Indigenous Australians with type 2 diabetes and other high risk populations.

Small participant numbers and the qualitative nature of this study may limit the generalisability of these results. Even with the current participant numbers, a significant amount of data saturation was attained with minimal new themes emerging from the data. It is suggested that anywhere from 5 to 50 participants are adequate for qualitative research depending on the heterogeneity of the population being sampled. Data saturation was also limited as this study was designed to be completed within a 6-month timeframe, hence only participants who had utilised 6-months of FGM could undertake the semi-structured interviews. Furthermore, qualitative research is often criticised for its validity and reliability, because compared to quantitative research, it is more prone to researcher bias [29]. However Indigenous people are disproportionately affected by type 2 diabetes, so it is imperative to identify strategies that can help improve their diabetes management, even within the limitations and scope of this study. Participants in this study had positive experiences of FGM use and it suggests that these technologies may help Indigenous people stay engaged with their diabetes care.

Recruitment occurred predominantly through Rumbalara Aboriginal Co-operative Medical Clinic. Those who attend this clinic do not represent all Indigenous Australians in the Greater Shepparton Region. Given the inclusion and exclusion criteria of the RCT, any findings from this study are limited to the scope of those participating in the RCT. There is a significant degree of heterogeneity across Australia amongst Indigenous people, so the responses towards new technologies may vary according to region. Therefore one cannot generalise the findings of this study across the continent. Even if participants numbers were greater, they represented the Greater Shepparton region, so these findings may not translate across Australia. Furthermore, participants in this study were recruited from a pilot RCT that is still recruiting. As such the findings in this study are not comprehensive, but provide cross-sectional qualitative data of experience of FGM at the time of writing. Given the extremely positive experiences of this new technology in an Indigenous population that has the potential to improve diabetes management independent largely via lifestyle changes, it was felt important to report these findings with minimal delay.

### Future directions

This was a small feasibility study that recruited participants from an ongoing RCT. Furthermore, any qualitative data serves to guide future study protocols that will quantitatively measure whether these positive experiences translate into improved glycaemic management. There are plans to expand this research into other Indigenous Australian communities, in metropolitan, regional and rural centres to gather the experiences of other Indigenous population groups outside the Greater Shepparton region.

## Conclusion

The use of FGM to assist individuals to monitor their glucose levels could be a novel approach to help manage diabetes in Indigenous communities in Australia. This qualitative study has shown that FGM is acceptable and provides potential benefits to Indigenous Australians living with T2DM in the Shepparton region.

### Supplementary Information

**Additional file 1.**

## Data Availability

The datasets use and/or analysed during this study are available from the corresponding author at reasonable request.

## Abbreviations

T2DM: Type 2 diabetes mellitus
FGM: Flash glucose monitoring
CGM: Continuous glucose monitoring
SMBG: Self-monitored blood glucose
RCT: Randomised control trial
BMI: Body mass index
HbA1c: Glycated haemoglobin
GP: General Practitioner
