# Supplementary material for: Feasibility and acceptability of the use of flash glucose monitoring encountered by Indigenous Australians with type 2 diabetes mellitus: initial experiences from a pilot study
Source: BMC Health Serv Res. 2023 Dec 8;23:1377. doi: 10.1186/s12913-023-10121-6 (PMC10704698; doi:10.1186/s12913-023-10121-6)
Supplement: Supplementary file 1 — Additional file 1. [file 12913_2023_10121_MOESM1_ESM.docx]

# Appendix

Comprehensive list of quotes obtained divided into themes developed from analysis of semi-structured interviews

| Acceptable |
| --- |
| “it was a very good program” – P1 |
| “it was alright. I wasn’t impaired by it or anything. So it was alright” – P2 |
| “it’s been great, the change over from using the, you know, pricking the fingers all the time, it’s been fantastic” – P3 |
| “it fits well, don’t even know it’s there” – P3 |
| “I’m in full support of it, you know for sure” – P3 |
| “quite good, you forget that it’s there” – P4 |
| “I reckon it was really good” – P4 |
| “I found that for myself it was beneficial” – P4 |
| “At first you knew that there was something there, but then you got use to it, it just grew with you and it become a part of you, with your diabetes, which was good” – P5 |
| “half the time you didn’t know it was there until you scanned it” – P5 |
| “it was a good machine, I’d recommend it, the machine” – P5 |
| “the whole thing is worthwhile” – P5 |
| “It never bothered me” – P5 |
| “at first you knew that there was something there, but then you got use to it, it just grew with you and it became a part of you, with your diabetes, which was good” – P6 |
| “forget that it’s there” – P8 |
| Negatives |
| “I tend to knock it off, other than that, it was good. I really liked it” – P4 |
| “You got to have umm…what do you call it, support with it, like tape” – P5 |
| “there might be a bit of redness around your arm, around where it is. But that goes away” – P5 |
| “just got to be careful where you lay and because you’ll get caught onto something and that something can just pull it off” – P8 |

| Convenience |
| --- |
| “I could monitor my sugar anytime I wanted to and I knew when it was high and when it was low” – P1 |
| “it was good, because I just needed to go like that [swipe] and I didn’t have to prick myself so it was wonderful” – P1 |
| “I’d just scan a lot and it was really good” – P1 |
| “because you can scan yourself and that’s one of the blessings because you’re not always able to stay here, you’ve got to go to doctor’s appointments, you’ve got to go down the street shopping, you’ve got to do other things. It’s good just to run it over and see if it’s high or low” – P1 |
| “monitor in the morning and lunch time and afternoon, night time” – P2 |
| “life a lot easier for me with that because it’s so simple and easy to use” – P3 |
| “I can record any time of the day, everyday, night, whatever” – P3 |
| “having that [device] has made it a lot easier” – P3 |
| “Well I actually liked it because you only had to scan it all the time so that was so much more easier than pricking your finger all the time and like if you were out and something you just go wave whereas you got to sit somewhere and you know, pick your finger and monitor and you’ve got everyone watching whereas that’s more discreet” – P4 |
| “I was more inclined to use, I don’t use the finger-prick much at all. But I did use that one” – P4 |
| “it helped me monitor my sugar late at night, during the morning” – P5 |
| “definitely found it much easier and good to take with me you know especially at work where I could just use it at lunch time” - P5 |
| “for travelling, if you’re travelling, it wasn’t a hassle of getting that bag and doing all that other stuff, it was just a matter of just scanning it and keep going” – P5 |
| “travelling long distance you know and just scanning it, you know it was easy as, instead of pulling up and then you know” – P5 |
| “just much more handy you know, having to only carry around that [reader] instead of the pouch that’s got the machine, the needles, it’s got the strips and all that” - P7 |
| “I’m glad that I didn’t have to prick myself every day. I’m glad that you can just scan it. That’s the best thing that ever happened to me” – P8 |
| Compared to SMBG |
| “your hands get so sore from getting pricked all the time” – P1 |
| “we are so sore in the fingers from being pricked you know. It’s a very cruelty, because they’re that sore and you think oh no, not again, you’re gonna get pricked again” – P1 |

| Information |
| --- |
| “was informed” – P4 |
| “I loved the information that is stored in it. So then it gives you an idea of how your sugars are going” – P4 |
| “makes you more inclined to try and do something. Because you can see, ooh hang on, what happened here? And then you can sort of rethink about what you do during the day” – P4 |
| “do it, because then they’d have an idea of what’s happening in their body. Because if you don’t. Ignorance I say, people think ignorance is bliss, but when it’s affecting your health so severely you really need to know and that’s what it did, it just showed you what you really needed to know” – P4 |
| “he’d benefit from it especially if you can see it. Because, Indigenous people are actually better at seeing things, it’s got more of an impact” – P4 |
| Influence on diet |
| “I’d wake up and do it to see what it was before breakfast and then I’d scan myself to see what I’m having after breakfast” – P1 |
| “I thought about what I ate a lot better. And I’d knock things back that” – P1 |
| “Cause us to think about what we were eating, what we were doing, trying to be exercising more” – P1 |
| “it stopped me eating a bit. So that was one thing about it. I was watching my food intake” – P2 |
| “Gave me insight into where I’m at. Yeah, stopped me from eating a lot of kabanas and chips. Some of my favourite foods” – P2 |
| “stop me from eating. It did stop me eating for quite a while” – P2 |
| “use it because it stops you from eating and makes you watch your food more” – P2 |
| “it makes you think about what you put in your mouth because it does show up and you can’t dodge no bullets, you can actually see it” – P4 |
| “You actually have to think about your diet, because once you put the food in you know it’s going to come up on it [device]. It makes you really think, whereas at the moment, I don’t think I just eat” – P4 |
| “Like I said you can see when it goes up and comes down and you’d ask, “oh how come”, and then I’d say “well, I wasn’t eating right, ooh okay” – P4 |
| “You’re constantly looking at the readings and then I think “oh, I’ve got good readings so I’m doing the right thing, so yeah it does, it does change what you eat” – P5 |
| “A better way of looking at it. You know what I mean? Especially if you’re going low and stuff, and too high, you’re like whoop, what did I have, what did I eat, what did I drink, that kind of thing” – P7 |
| Lack of understanding |
| “I don’t’ understand it at all, but I know that it’s you know, it’s for professional people to read it, and they can understand over a period of time” – P3 |

| Support |
| --- |
| “it’s been very very good because people have been wonderful, they’ve come and helped and supported me, [DNE]’s come over from over there and put the thing in my arm whenever it needed to be done” – P1 |
| “I’ve enjoyed the people I’ve worked with and very grateful for the people that have helped and supported me in trying to bring something new to help and support diabetes” – P1 |
| “So everybody worked very very well together and supported me which is wonderful” – P1 |
| “usually one of the nurses will do it for me, it’s a lot easier” – P3 |
| “happy to come in, always good to come in, because you catch up with people, like the relatives and so on, because I’m retired of course. So I don’t mind at all” – P3 |
| “I could probably do it but, yeah, just come in and have a chat” – P3 |
| “it was just good and when you’re around people you know, and even with that [device] on it just brought sugars down because you know you’re with good friendly people and that” – P5 |
| “thank you for giving me the opportunity anyway and being support to me as well” – P5 |
| “it was good to have support, people that are around that communicate and help” – P6 |
| Local Health Provider |
| “First time they’d seen it” – P2 |
| “But normally he’ll leave it up to the professional people” – P3 |
| “They knew of the machine, but they didn’t ask questions about what does it do” – P5 |

| Community |
| --- |
| “they’ll say ‘oh gee how can I get one’ you know, especially the ones that have diabetes” – P3 |
| “I think they’re fascinated by the technology of reading their sugars and the ones which that have diabetes ‘where’d you get that, where’d you get that, how’d you get that” – P3 |
| “Ooh that’s good, that’s cool, yeah.” So yeah they all liked it” – P4 |
| ““I’ve never seen anything like that” but it was just a new device and they, most people understood, diabetes and that” – P5 |
| “they say you know, they should bring out more technology or something for diabetes” – P5 |
| “everyone was like, check your sugar, check your sugar.” – P7 |
| “I’d have the little blole…he thinks it’s cool, so he’s always on my back, checking, checking, checking” – P7 |
| Negatives |
| “people do judge you, because you’re Aboriginal, you’ve got diabetes” – P5 |
| “it’d depend on if other people seen that I’ve got it” – P5 |

| Cost |
| --- |
| “But I know it’s very expensive to go and buy now because that was a program that I was on and I really can’t afford it” – P1 |
| “but it’s not cheap. It’s expensive. But it’s a new program and it’s really helpful. So I hope the government will continue to help and support people high sugar” – P1 |
| “I hope it goes all around Australia, and the government will support it” – P1 |
| “you can buy it, you can put it through yourself, but we haven’t got the money love because we’ve got to pay 350 a week here and we’re not left with a lot of money” – P1 |
| “A hundred dollars each hey? Expensive…yeah. Yeah, expensive little program” – P2 |
| “I’d tell them it’s a good thing to have. But paying for it, no. Not a hundred dollars a go. For free yeah” – P2 |
| they’re pretty expensive to buy. So I don’t know what’s going to happen when the times up and you’ve got to buy your own” – P3 |
| “Yeah if they can get that subsidised, well it would be great for diabetics to do their recordings” – P3 |
| “it was good. I really liked it. I wish I could go in to the chemist and say could you give me one of those please” – P4 |
| “if it was cheaper, I’d continue to get it you knowm but I just don’t know that spending that every fortnight you know…might hurt the budget a little” – P7 |
